# Influence of the In-Fill Pattern of the 3D Printed Building Wall on Its Thermal Insulation

**DOI:** 10.3390/ma16175772

**Published:** 2023-08-23

**Authors:** Paweł Dziura, Marcin Maroszek, Mateusz Góra, Magdalena Rudziewicz, Kinga Pławecka, Marek Hebda

**Affiliations:** Faculty of Materials Engineering and Physics, Cracow University of Technology, Warszawska 24, 31-155 Kraków, Poland; pawel.dziura@doktorant.pk.edu.pl (P.D.); marcin.maroszek@doktorant.pk.edu.pl (M.M.); mateusz.gora@doktorant.pk.edu.pl (M.G.); magdalena.rudziewicz@doktorant.pk.edu.pl (M.R.); kinga.plawecka@pk.edu.pl (K.P.)

**Keywords:** 3D concrete printing, thermal conductivity, numerical simulation, geometry

## Abstract

The intensive development of 3D Concrete Printing (3DCP) technology causes constantly increased its share in the construction sector. However, in order to produce products with controlled properties, optimization of the technological process is still required. Automation of production based on additive manufacturing methods streamlines the process by comprehensively manufacturing building components that meet, among others, strength, visual, and insulation requirements. Moreover, the possibility of using computer simulations to assess the properties of the designed elements allows for a multitude of analyzed versions of the constructed partitions, which can be verified at the design stage. Thanks to such an opportunity, the process of designing building elements can be significantly improved. The article presents results related to the assessment of the level of thermal insulation of products that can be produced by additive technology, depending on the applied spatial geometry of the vertical partition and the amount and type of materials used. Eight original solutions of in-fill pattern were designed, for which both Finite Element Method (FEM) computer simulations of thermal conductivity and experimental measurements of thermal conductivity of samples were performed. On the basis of the obtained results, both the correctness of the simulation results for the various analyzed materials and their consistency with the practical results were found. Depending on the investigated geometry, for samples of the same dimensions and using the same material, the differences in the U-factor obtained by FEM analysis amounted to 61%. The best solution from the investigated spatial geometries of the vertical partitions has been indicated. The U parameter in the variant with the best thermal insulation was 0.183 W/m^2^K, which meets the requirements of Polish construction law. The issues discussed in this work can be the basis for the selection of the best solution possible for practical use during the production of building walls using the 3DCP method fulfilling the guidelines of applicable standards. Furthermore, they can be used as a tool for optimizing geometry in terms of energy savings and reducing waste production by both engineers developing 3DCP technologies and architects using innovative techniques for manufacturing building structures.

## 1. Introduction

Three-dimensional printing technology is now a widely known additive manufacturing method of elements from many different materials. The constantly growing range of applications, easier access, and thus greater interest in this technique allowed for its huge development in recent years. Currently, the most widespread is printing from polymers and metals, which enables the production of elements of almost any shape. In many industrial plants, this technology is a supplement to conventional machining techniques [1,2]. Its new field of application is the medical or space industries. Chen et al. present promising results in the development of tissue printing technology; however, the complicated and lengthy process of testing and approval for use means that this technique still remains in the realm of prototypes [3]. The possibilities of 3D printing are also increasingly used in the construction sector known as 3DCP. Its intensive development in recent years is, among others, a response to the market demand, which are problems with excessive waste production and the lack of qualified workers [4,5,6]. In the construction industry, 3D printing technology is perceived as environmentally friendly due to the freedom of design, automation, less waste generation, lower consumption of raw materials, and lower labor costs [7]. These benefits also include the reduction in production costs resulting from the elimination of formwork and the reduction in workload, thus shortening the duration of projects, which translates into an increase in the competitiveness of modern additive technologies in relation to traditional methods of manufacturing building elements [8]. This makes the interest in this technology not only related to the research area but also to the industrial sector as a technology that allows the implementation of innovation and competitiveness of solutions in relation to competition [4,9,10]. An additional advantage of this technology may be solutions that allow the construction of building partitions with individual geometry and from materials ensuring both mechanical strength and high thermal insulation parameters. In the construction industry, Portland cement is still the most commonly used material in the world, which is characterized by resistance to fire, rust, and decay, as well as flexibility in forming and shaping [11,12,13,14]. The intensive use of this material has been discussed by many researchers on its impact on the natural environment in the last decades because the production of cement has a negative impact on the environment through the use of large amounts of raw materials and high energy requirements [15,16]. These factors translate into a large amount of carbon dioxide released into the atmosphere, and thus global warming [12,13,17]. Therefore, it is necessary to reduce the amount of Portland cement used in the construction sector, which can be achieved by developing new alternative materials, e.g., geopolymer binders, or by producing building elements based on geometries that require less material and reduce the generation of waste. The 3DCP method gives almost endless possibilities in terms of the geometry of the created elements. Its principle of operation consists in transforming a 3D solid saved in a digital file into a physical spatial structure [18,19]. Based on these features of the technology, it is possible to develop optimal geometries of vertical partitions of buildings, which, apart from lower demand for building material, will meet the requirements of both construction and thermal insulation layers [20,21]. Obtaining a thermal partition of the building with appropriate properties is necessary both for economic reasons, by reducing the costs of heating and cooling utility rooms, and for environmental reasons, in order to minimize energy losses. Moreover, actions aimed at improving the thermal insulation of building partitions and reducing the overall energy consumption for heating are required in accordance with the objective of the European Energy Efficiency Directive, which is the climate neutrality of buildings by 2050 [22]. According to the latest construction requirements for the thermal protection of buildings in Poland (WT 2021), the maximum value of the heat transfer coefficient Uc(max) for vertical partitions is 0.20 W/(m^2^K) [23]. Such requirements mean that each new building must be carefully protected against loss of thermal energy through all external partitions. In order to achieve such a level of thermal protection, it is necessary to use an appropriate thickness of insulating materials, which are traditionally installed as an additional layer on external walls [22]. Such solutions, however, generate a significant amount of waste and require a lot of work by qualified employees [21]. In addition, when insulating vertical partitions with an external insulating layer, the outline of the building itself increases significantly, which also often causes problems and is not possible in all places. Using the possibility of 3D printing to produce a vertical partition having both the parameters of the load-bearing wall and the insulation layer would allow for a significant reduction in the amount of work, the amount of waste, and thus the costs of building production [24]. However, the production of vertical partition of a building using 3DCP requires its prior full modeling. During this operation, it is necessary to take into account not only its external dimensions but also the internal structure of the filling, so as to ensure adequate stability during the printing process, the required load capacity of the structure, and the assumed thermal insulation [5,25]. In addition to the advantages of creating arbitrary geometries with internal voids and using different materials during detail printing, the capabilities of 3DCP technology can also positively affect a wide variety of building features [26,27]. This is being studied in detail by researchers, who are testing the parts produced with this technology from many different aspects. Prasittisopin et al. verified the thermal and acoustic insulation properties in walls printed from material with different compressive strengths and textures of the printed panels. They showed that blends with lower compressive strength, which have more pores, insulate better. Similarly, wall structures having more pores or air voids show better thermal insulation [28]. Advanced numerical analyses, on the other hand, were carried out by Suntharalingam et al. which determined the level of fire performance of printed walls, depending on the wall structure as well as the insulation used [29]. Intensive work on the development of 3DCP is also underway in terms of reducing the materials and energy consumed in the printing process, which undoubtedly benefits the environment [30,31].

The article presents the results of a comparison of computer simulations and experimental measurements related to 3DCP printing of vertical partitions made of a combination of concrete and insulating material, so that, in contrast to conventional construction, the Umax parameter of the partition was reduced without increasing the outline of the building.

## 2. Materials and Methods

In order to achieve the best possible results of partition insulation, it is necessary to use such an internal geometry that will maximize its thermal resistance. Insulating materials such as foams or wools, whose low thermal conductivity reduces energy losses, are perfect for this purpose. However, the partitions of buildings are also to ensure adequate load-bearing capacity, for which it is necessary to use solid materials such as concrete, whose insulating properties are much weaker. Therefore, it is important to prepare the internal geometry of the partition in a way that maximizes the energy flow path through the solid material of the partition. Such a solution will also reduce thermal bridges in places where the internal and external surface of the partition is connected to a well-conductive material. The presence of such connectors is, in turn, determined mainly by the technological requirements of 3DCP, but also by the need to close the space inside the partition before filling it with insulating material in order to ensure adequate load-bearing capacity of the partition produced [5]. At this point, it should also be mentioned that the performance of the partition is also influenced by the surface itself, which in the vast majority of 3DCP printing cases has a characteristic texture in the form of high roughness and fine grooves between layers, which is particularly evident in 3DCP analysis based on architectural perspective [32,33]. However, this is not a rule because there are solutions on the market in which the print head is constructed in such a way as to smooth the lines of the applied material, getting rid of the above imperfections [34]. During the implementation of these studies, it was decided to omit the aspect of wall roughness and rendering the texture of layering. Based on the above assumptions and additionally taking into account the reduction in production costs and minimization of waste, the evaluation of thermal insulation parameters was carried out using a computer FEM simulation for eight original concepts of partition geometry. The numerical simulation was carried out in the SolidWorks 2022 program with the use of the Simulation module, which enables calculations of heat transport in a given system. Each concept was created taking into account the technological possibilities of 3DCP printing. The base element was a prepared architectural 3D model of the partition with overall dimensions: 1000 mm × 700 mm × 430 mm. This model also contained information about the component materials, which included normal strength concrete as a load-bearing part and polyurethane (PUR) foam as a filling and thermal insulation of the partition. The simulation module required supplementing the data on the boundary conditions of the performed analysis and the method of performing the calculations. Verification of the numerical results was carried out on a reduced scale using the HFM 446 Lambda Eco-Line heat flow meter by Netzsch, on samples with dimensions of 200 mm × 200 mm × 50 mm. Such a small thickness of the sample made it impossible to produce it using the 3DCP method. The minimum possible nozzle size in 3DCP technology is usually larger than 20 mm, as it depends on the material used and its aggregate fraction components. Therefore, all the tested variants of the geometry were rescaled while maintaining all proportions. To make samples on a smaller scale, the Fused Deposition Modeling (FDM) 3D printing method was used, which allowed to obtain the required properties. Due to the verification of only the geometry of the partition, the impact of the printing method on the obtained results was considered negligible. The numerical determination of thermal conductivity levels in all geometry variants was based on prepared architectural 3D models and material data. In the simulations, normal strength concrete was the material for the construction of printed building elements. The printed geometries were to meet the requirements of transferring compressive stresses generated by the weight of the entire building and to give the final shape to the resulting elements. The voids created inside the partition were filled with an insulating material—PUR foam, in order to improve the thermal insulation properties of the designed partitions. Table 1 shows the properties of the materials used in the computer simulation. The calculations were also supplemented with an analysis of the configuration, in which the carrier material was polylactic acid (PLA) used in the verification process.

Models for testing on the HFM 446 Lambda Eco-Line heat flow meter were made in FDM technology on the Bambu Lab X1 device. The main printing parameters were presented in Table 2.

**Figure 1:**
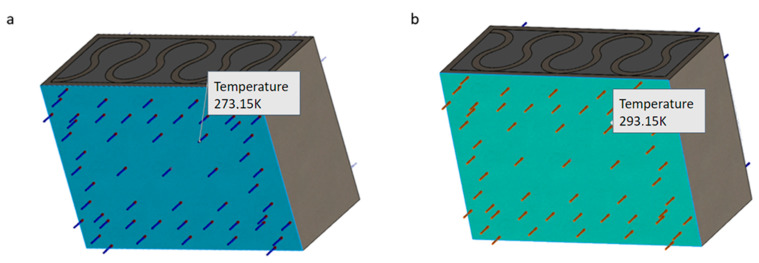
Model presenting the influence of temperature on the sample: (**a**) 273.15 K on the cold side and (**b**) 293.15 K on the hot side.

The FEM thermal simulations were performed as a stationary heat flow (steady-state conditions/heat transfer) through a segment of the designed vertical partition of the building. In addition to preparing the 3D model, before starting the simulation, it was required to define the assumptions and boundary conditions of the observed phenomenon. The considered case was described using the convection phenomenon (Convection Thermal Load function—Simulation SW 2022 module), for which the following parameters were set: external temperature (cold side) 273.15 K and external film coefficient 25 W/(m^2^K) (Figure 1a); internal temperature (hot side) 293.15 K and internal film coefficient 7.7 W/(m^2^K) (Figure 1b) [20,28,29]. The values of the coefficients depend, among others, on the type of convection and the fluid in contact with the partition. Natural convection, which usually occurs inside the rooms, has lower values than forced convection, operating from the outside of the partition, where the wind forcing air movement intensifies the heat flow process.

In order to obtain repeatable and error-free results, high quality mesh was used, the parameters of which for one of the geometries are listed in Table 3. The average aspect ratio value was in the range of 1–3, while the maximum aspect ratio was less than 100. A mesh of such quality allowed to obtain accurate and repeatable results of simulations of processes related to heat transport through the partition [35].

After the simulation, the average heat flow through the entire surface of the wall was determined, which was used for further calculations. The obtained result was divided by the difference of the set temperatures, thanks to which the coefficient of thermal conductivity U was obtained, according to the Equation (1). In all measurements, a temperature difference of 20 K was assumed.

(1) $$U=\frac{Hf_{AVG}}{\Delta T}\;\;\;\;\;\;\;\left(\frac{W}{m^{2}K}\right)$$

*Hf_AVG_*—average heat flow (W/m^2^)

Δ*T*—temperature difference (K).

## 3. Results and Discussion

Nowadays, generally, the created structures have fillings where the opposite walls of the partition were connected with each other with perpendicular lines [20]. This solution adversely affects the 3D printing technology as well as the thermal insulation of the wall, due to the minimization of the length of the thermal bridge between the hot (internal) and cold (external) sides. Therefore, in the presented solutions, the geometrical concept of the partition was designed in such a way as to extend the connectors between the internal and external surfaces of the partition as much as possible. These elements are structurally necessary because they are designed to increase the load capacity and stiffness of the product. On the other hand, extending these sections can significantly reduce the occurrence of thermal bridges resulting from intensive heat conduction through the carrier material, which has low insulating properties. Table 4 presents the eight author’s concepts of the geometry of the vertical partition. Each of the analyzed solutions was characterized by the same overall dimensions (1000 mm × 700 mm × 430 mm) and the same path thickness of 30 mm. The visualizations were supplemented with information on the volumetric share of the load-bearing material in the analyzed samples.

In the first concept, the reinforcement printed between the external and internal sides of the partition takes the shape of a triangle, which is filled with insulating material. These fasteners are set at an angle of 65 degrees to the external surfaces. In the second concept, the geometry of the printed reinforcement adopts a shape similar to that of concept 1, while roundings have been additionally used to optimize the movement of the printing head and increase the length of joining the extreme surfaces. The reinforcement path has been tangentially connected to the path of the outer surfaces. The third and fourth concepts correspond, respectively, to the first and second variants in terms of cross-section. In a section parallel to the outer surface, however, the partitions change their arrangement at an angle of 70 degrees to the base. Such geometry is made by shifting each of the layers of the printed reinforcement perpendicularly to the direction of component growth. A visualization of the differences between these geometries was presented in Table 2 in the column “cross-section parallel to the external surface of the partition” for variants 3 and 4. The fifth concept consists of a strongly corrugated filling, which was created on the basis of externally tangential circles with a diameter of 200 mm. The intention of this concept was to maximize the length of the heat flow path through the carrier material. In concept 6, from the inside of the building, a rectangular filling was designed to cover 30% of the width of the partition, and the reinforcement structure from the outside takes the shape of a triangle. Concepts 7 and 8 consist of two equal-sized chambers, each with a triangular geometry. However, in concept 7 the triangles are arrayed and in concept 8 they are mirrored.

**Figure 2:**
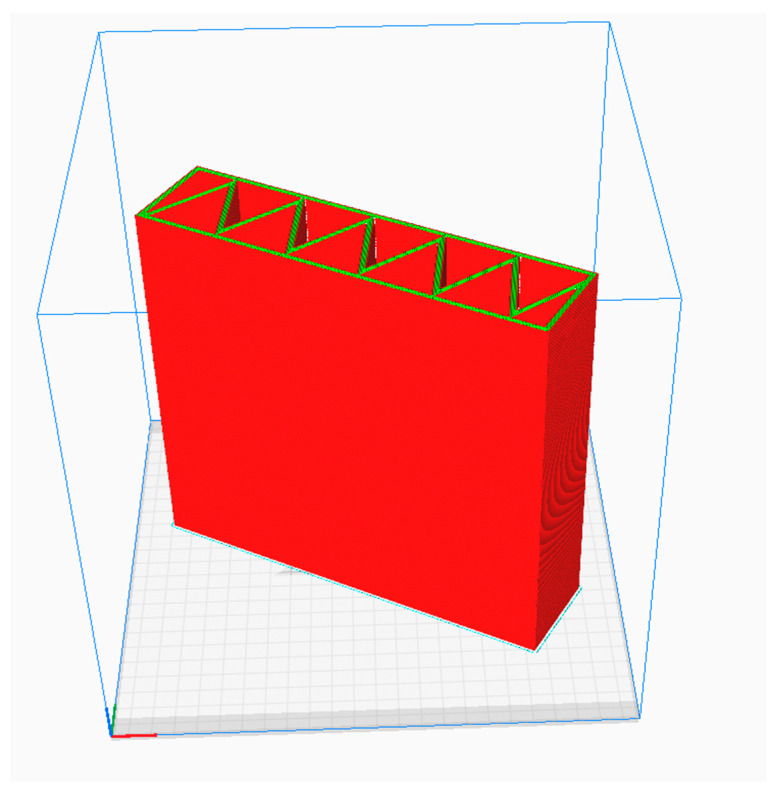
Orientation of the building partition during printing in FDM technology—preview of the program for preparing the printing process.

Due to the fact that the process of producing partitions in the 3DCP technology will take place in a vertical orientation, the FDM models were printed in a similar way (Figure 2).

**Figure 3:**
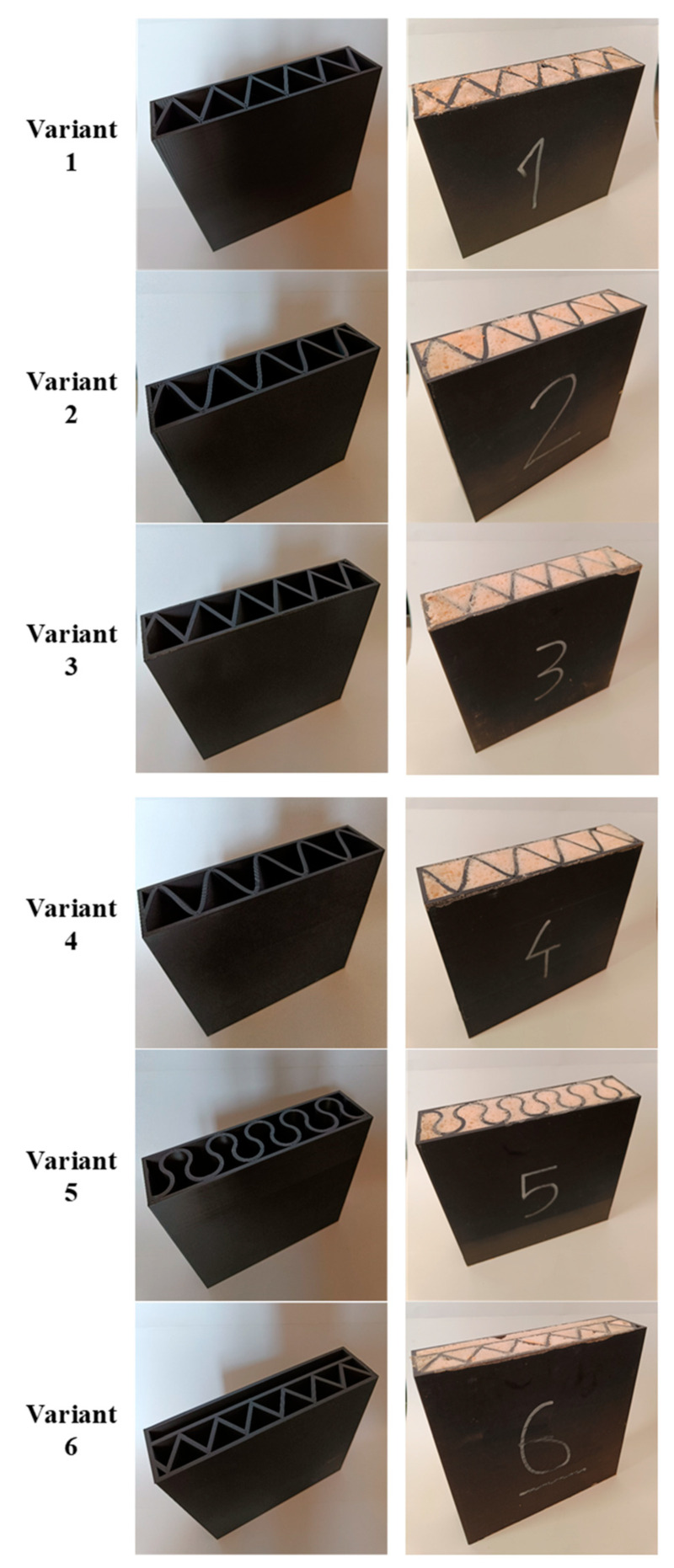

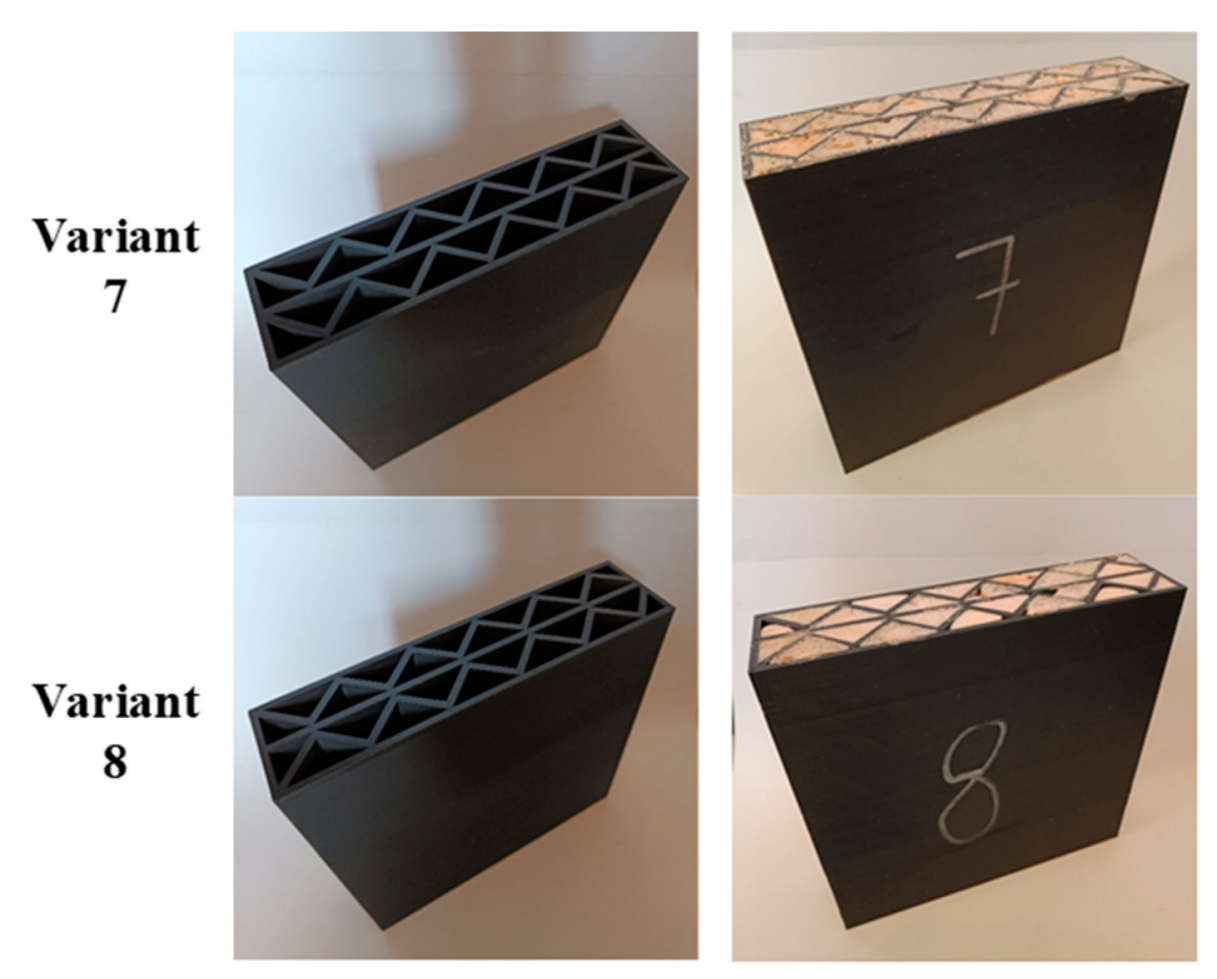
View of variants of partitions printed in FDM printing technology from PLA (**left**) and samples ready for testing after filling with insulating material—PUR foam (**right**).

Examples of samples calibrated and printed with PLA are shown in Figure 3. The printed samples were filled with an insulating material, i.e., expanded PUR foam. The effect of the produced samples was shown in Figure 3.

### 3.1. Thermal Simulation Analysis

**Figure 4:**
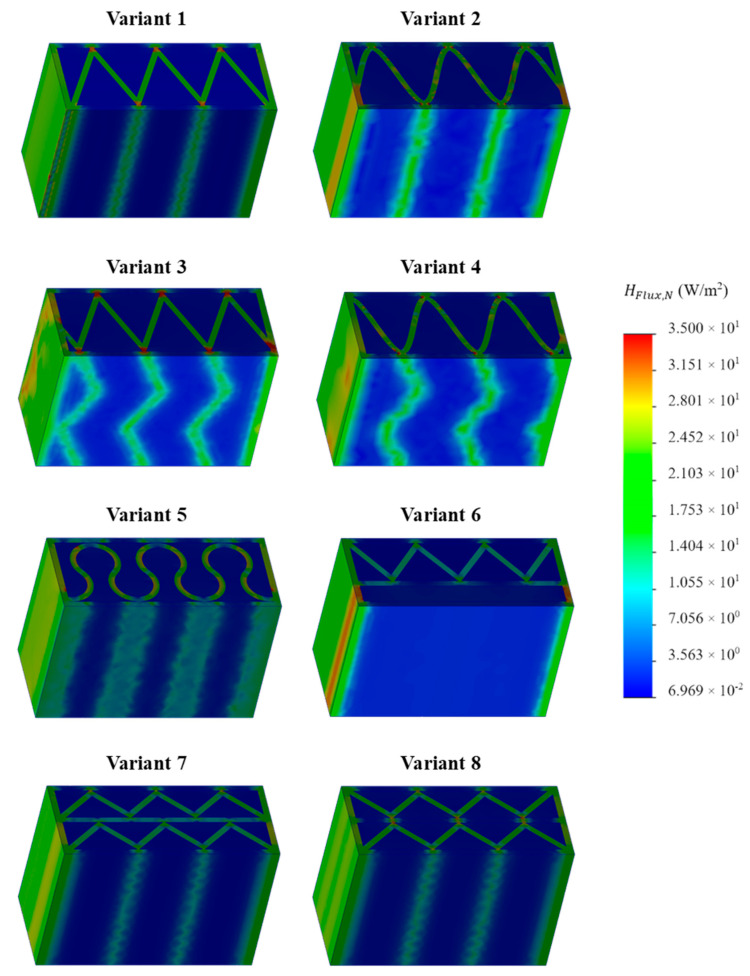
Results of thermal simulation of heat flow for individual variants of the tested samples, assuming that the load-bearing material was concrete.

**Figure 5:**
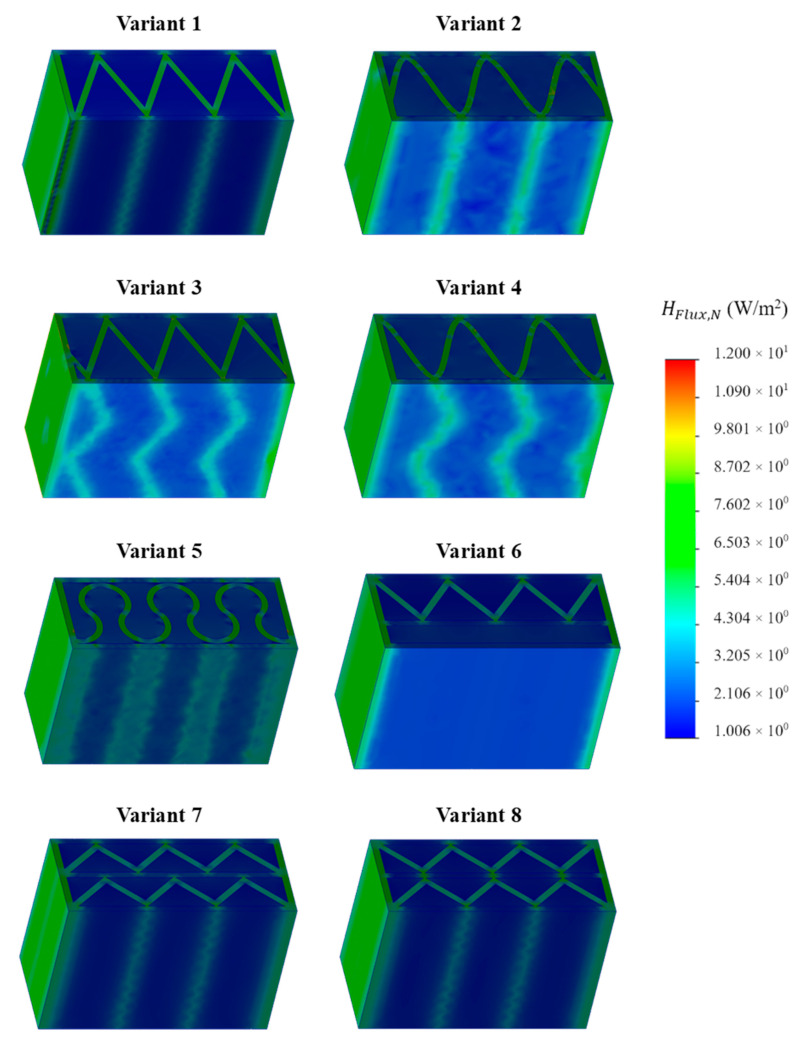
Results of thermal simulation of heat flow for individual variants of the tested samples, assuming that the load-bearing material was PLA.

FEM computer thermal simulation allowed to obtain the distribution of heat flow values in the space of the analyzed sample. The analyses were performed with the assumption that the load-bearing material was concrete (Figure 4) or PLA (Figure 5). If a point or space of the obtained simulation was closer to red, it meant a greater heat flow (energy loss) in this place of the sample. On the other hand, if the color was closer to navy blue, the less heat flow occurred, which means more effective insulation.

Based on the obtained simulation results, it was possible to visualize and indicate the exact locations of thermal bridges. PUR foam, as an insulating material, resists the flow of heat in a very effective way. Therefore, in the developed variants of the partition geometry, it was crucial to make the most of its advantages and to reduce the occurrence of thermal bridges as much as possible. Due to this, it was important to obtain the longest heat flow path through the carrier material. For each simulated concept, the average heat flow through the outer and inner sides of the partition was determined, and then the arithmetic mean was calculated from them. The results of the calculations were summarized and presented in Table 5.

**Figure 6:**
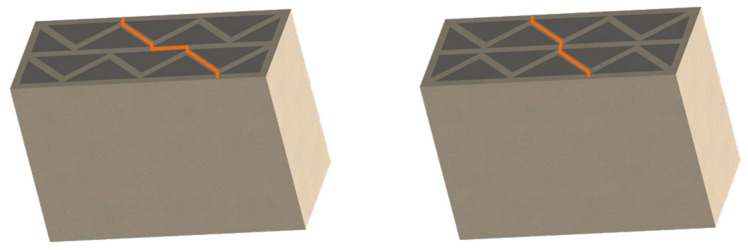
The distance along the load-bearing material between the inner and outer surface of the partition for variants 7 and 8. The orange line represents the shortest distance through the in-fill patterns.

On the basis of the obtained results, a significant difference in the heat flow, and thus also in the U-value, was found between the two analyzed material configurations. This effect was the result of a significant difference in the thermal insulation properties of both load-bearing materials, where the thermal conductivity coefficient for PLA is more than 70% lower than for concrete. Ignoring this factor and focusing on the comparison and analysis of individual partition geometry variants, a visible and repeatable hierarchy of the analyzed solutions was found. Among the tested samples, the lowest heat transfer coefficient was obtained by concept no. 6, with the result of UC 0.1827 W/m^2^K for concrete and UP 0.1048 W/m^2^K in the configuration with PLA. The wall with a triangular filling with an additional narrow space filled with insulating material was certainly distinguished by the elimination of the connectors of the internal and external surface of the partition in part of the cross-section. Their absence limited the formation of thermal bridges, and thus limited losses. The essence of these loss sources was evident in the U-factor of the best variant, which was, respectively, 23% and 10% lower for concrete and PLA compared to the second-best result. Variant 6 was a geometric solution that most closely resembles the combination of a hollow brick and a layer of polystyrene known from conventional construction, with the advantage that in 3D printing technology, free spaces in the load-bearing zone can be insulated as part of one automatic process. Analyzing further simulation results, the second-best geometrical concept in terms of thermal insulation was variant number 7, in which two rows of ribs with an extended distance between the extreme surfaces of the partition were used. The U-value for this variant was UC 0.2384 W/m^2^K and UP 0.1161 W/m^2^K, for concrete and PLA, respectively. These results are, respectively, 4 and 1.5% lower than concept no. 8, which also had two rows of ribs but had a shorter distance between the extreme surfaces—the difference was shown in Figure 6. The next results were for the pair of concepts 2 and 4. They were similar to each other in terms of the geometry of the cross-section, while in variant 4, an additional inclination of the walls along the increment of the partition was also used. This solution turned out to have a negative impact on the level of insulation. A similar situation occurred in the case of variants 1 and 3. In the conducted study, the lowest insulation parameters were obtained by variant no. 5, which obtained an almost twice as high coefficient in the variant with concrete and an almost 30% worse result in the PLA configuration.

### 3.2. Micro-Scale Wall Testing

The results of thermal resistance for all analyzed variants of the samples and the heat transfer coefficient U calculated on this basis (Equations (2) and (3)) were presented in Table 6. As in the case of simulation results, also in the verification test, the best results in terms of thermal insulation were obtained by samples designated as variant no. 6 and no. 7. The values of the U coefficient of these partitions reached high values, i.e., 0.899 and 1.038 W/m^2^K, respectively. However, the analyzed samples were only 50 mm thick, and this has a direct impact on the thermal resistance and thus the U parameter. Therefore, the achievable U parameter was also estimated for a partition with a thickness of 430 mm, i.e., equal to that assumed in the computer simulation.

(2) $$U=\frac{1}{R}\;\;\;\;\;\;\;\left(\frac{W}{m^{2}K}\right)$$

(3) $$R=\frac{d}{\lambda }\;\;\;\;\;\;\;\left(\frac{m^{2}K}{W}\right)$$

where:

R—thermal resistance (m^2^K/W),

d—thickness of the wall (m),

λ—thermal conductivity (W/mK).

Considering that the target wall will be much thicker, the U-value will oscillate around 0.10–0.14 W/m^2^K. It should be noted that such coefficient values, although they prove a high degree of thermal insulation, will not be able to be obtained in the final implementations, due to the PLA material used. However, these results can be used to verify the simulation.

**Table 6:** Thermal resistance, thermal conductivity, and thermal transmittance of samples depending on the tested variant of partition geometry.

Version No.	Thermal Resistance R (m^2^K/W)	Thermal Conductivity λ (W/mK)	Thermal Transmittance of Wall Systems U_T_ (W/m^2^K)	Thermal Transmittance of Wall Systems U_T_ (Wall Thickness 430 mm) (W/m^2^K)
1	0.8578	0.0584	1.168	0.1358
2	0.9050	0.0555	1.109	0.1290
3	0.8593	0.0586	1.171	0.1362
4	0.8915	0.0563	1.126	0.1309
5	0.8642	0.0579	1.158	0.1347
6	1.1391	0.0449	0.899	0.1045
7	0.9636	0.0519	1.038	0.1207
8	0.8671	0.0579	1.158	0.1347

**Figure 7:**
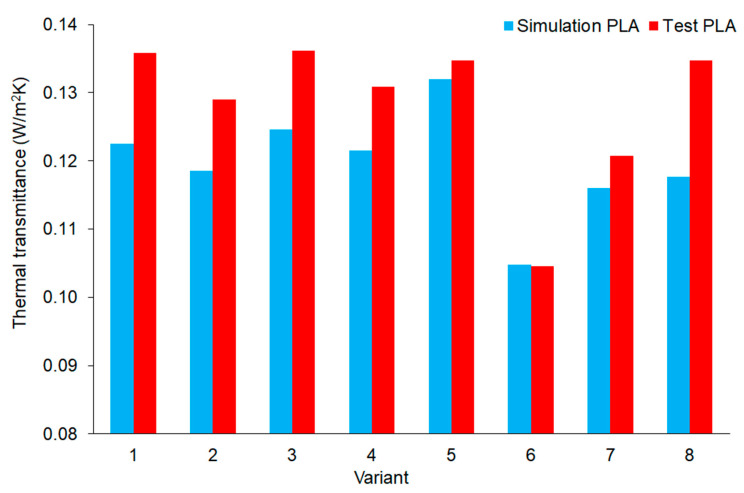
Comparison of computer simulation results and recorded results from thermal measurements, depending on the tested variant of the sample geometry, for the load-bearing material PLA.

In order to compare the results obtained from the simulation with the experimental data, the values of the U parameter were summarized and presented in Figure 7. On the basis of the analysis of the obtained results, it was generally found that the simulations were consistent with the measurements and that the sequence of individual variants was maintained in terms of their thermal insulation properties.

The results of the thermal measurements carried out also showed some differences in relation to computer calculations. The position of the result of variant no. 5 and variant no. 8 changed significantly, while the sequence of variants showing the best thermal insulation results was maintained (Table 7). The order in variants 1–4 was similarly maintained, where the effect of no increase in the thermal insulation of the samples was confirmed with the introduction of the inclination along their increment during printing.

**Figure 8:**
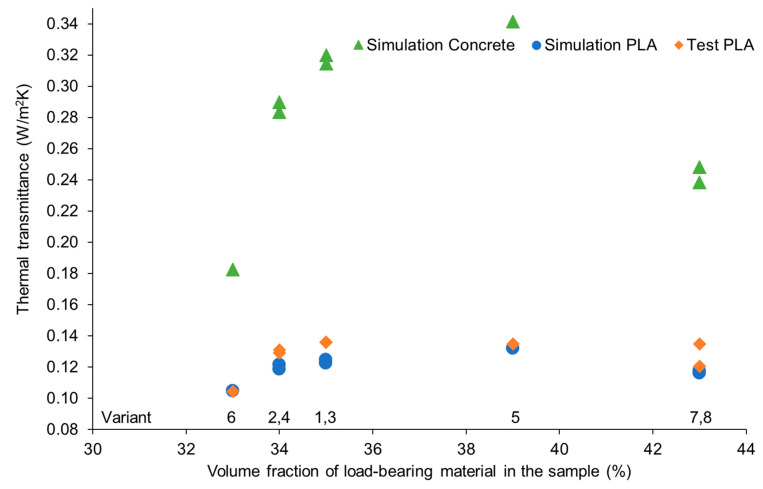
Heat transfer coefficient (measured or determined on the basis of simulation) depending on the type and volumetric share of the load-bearing material and the geometry of the analyzed partition.

Based on the results of the heat transfer coefficient through the proposed variants of partitions, a significant difference in its value was found depending on the geometry of the sample. The trends of the tested geometries were maintained regardless of the type of material used (Figure 8). Moreover, a decrease in the U-factor was observed for variant no. 7 and no. 8, despite the higher share of the load-bearing material necessary to make these geometries.

## 4. Conclusions

The internal geometries of the walls described in this article have a high potential for use in the construction industry for the production of building partitions. The properties of 3D printing technology and the vast possibilities it offers in terms of customization suggest it can be used for the automatic production of walls with parameters that meet the requirements of construction standards.

The results of the study clearly indicate that the internal geometry of the partition has an impact on thermal insulation. The geometrical concept of the investigated partitions was designed in such a way as to extend the connections between the internal and external surfaces of the partition to maximalize the length of the thermal bridge and therefore improve the thermal insulation of the wall. During the computer simulation, eight samples made of the same materials (concrete + PUR foam) and with the same external dimensions (1000 × 700 × 430 mm) with the difference in the in-fill pattern were analyzed. Scaled samples, made of PLA as a load-bearing material + PUR foam as insulation, were used for the experiment. The results showed a difference of 61% between the specimen with the highest and the lowest value of the heat transfer coefficient U for the simulation and 23% for the values achieved during the investigation.

Experimental verification of simulation studies confirmed the potential of the considered variants of partitions, marked with numbers 6 and 7, in terms of thermal insulation. The heat transfer coefficient U for variant number 6, where concrete was used as the load-bearing material, was 0.183 W/m^2^K. The geometry of such partition meets the construction requirements for thermal insulation, the limit value of which is 0.2 W/m^2^K.

The obtained results also confirm the correctness of using simulation as a tool for initial analysis and narrowing down the number of tested variants of samples in subsequent iterations of research.

The continuation of the results presented in this work will be the analysis of a selected variant of the partition geometry in full scale, made by 3DCP from materials adapted to the production of building elements. In addition, an important issue will also be the development of research on the simulation and verification of building structures in terms of mechanical strength, which is very important in the construction sector. Obtaining an advanced tool for the initial and comprehensive computer analysis of building elements will translate into the possibility of verifying complex versions of such elements at the design stage, and this will strengthen the competitiveness and potential of 3DCP technology.

## Figures and Tables

**Table 1:** Properties of materials used in an investigation of the concept of partition insulation geometry.

Material Property	Normal Strength Concrete	PUR Foam	PLA	Units
Elastic Modulus	32,000	2200	2950	N/mm^2^
Poisson’s Ratio	0.2	0.32	0.33	-
Shear Modulus	13,300	830	1200	N/mm^2^
Density	2460	16.02	1250	kg/m^3^
Tensile Strength	3	0.79	45	N/mm^2^
Compressive Strength	25	0.64	60	N/mm^2^
Yield Strength	-	-	35	N/mm^2^
Thermal Expansion Coefficient	10^−5^	6.5 × 10^−5^	7 × 10^−5^	K^−1^
Thermal Conductivity λ	0.5	0.027	0.13	W/(mK)
Specific Heat Capacity	750	1.2	1800	J/(kgK)

**Table 2:** FDM printing parameters.

Parameter	Value	Units
Nozzle diameter	0.6	mm
Layer Height	0.32	mm
Wall line count	6	N/A
Infill	100	%
Printing Temperature	210	C
Bed Temperature	60	C
Printing Speed	50	mm/s

**Table 3:** Mesh parameters for simulation processes.

Details Mesh Type	Solid Mesh
Mesher used	Blended curvature-based mesh
Jacobian points for high quality mesh	16 points
Max element size	38.0215 mm
Min element size	12.6737 mm
Mesh quality	High
Total nodes	88,252
Total elements	48,751
Maximum aspect ratio	18.272
Percentage of elements with aspect ratio < 3	97
Percentage of elements with aspect ratio > 10	0.107

**Table 4:** Visualization of variants of the analyzed geometries of the vertical partition with information on the volume fraction of the load-bearing material.

Samples Designation	Projection	Cross-Section	Cross-Section, Parallel to Wall Surface	Volume Fraction of Load-Bearing Material in the Sample
Variant 1	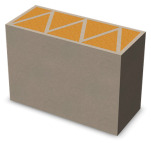	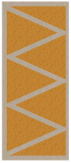	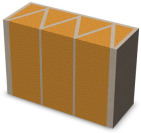	35%
Variant 2	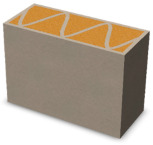	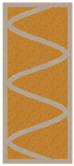	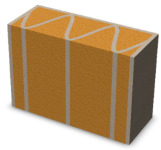	34%
Variant 3	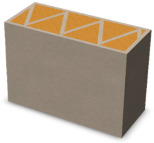	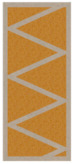	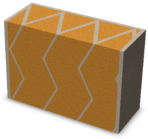	35%
Variant 4	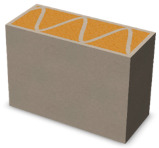	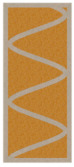	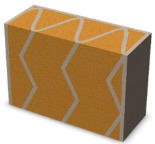	34%
Variant 5	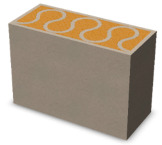	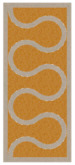	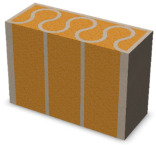	39%
Variant 6	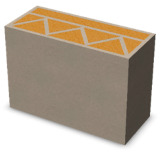	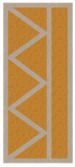	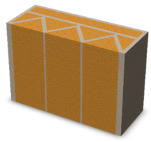	33%
Variant 7	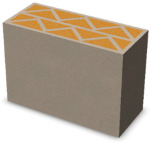	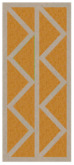	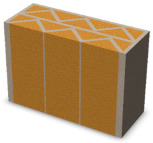	43%
Variant 8	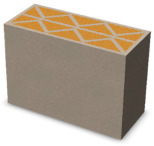	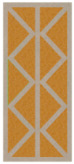	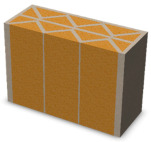	43%

**Table 5:** Values of heat flow depending on the geometry of the tested partitions and the type of carrier material.

Variant No.	Concrete		PLA	
	Avg. Heat Flux $Hf_{AVG,\;C}$ (W/m^2^)	Thermal Transmittance of Wall Systems U_*C*_ (W/m^2^K)	Avg. Heat Flux $Hf_{AVG,\;P}$ (W/m^2^)	Thermal Transmittance of Wall Systems U_P_ (W/m^2^K)
1	6.289	0.3145	2.451	0.1225
2	5.666	0.2833	2.370	0.1185
3	6.398	0.3199	2.492	0.1246
4	5.794	0.2897	2.431	0.1215
5	6.830	0.3415	2.639	0.1320
6	3.654	0.1827	2.096	0.1048
7	4.768	0.2384	2.321	0.1161
8	4.962	0.2481	2.354	0.1177

**Table 7:** Comparison of U-factor order recorded from the thermal measurements and obtained from computer simulation depending on the tested variant of the sample geometry, and the load-bearing materials. The green color represents the best results in each category, while the more red the shade, the weaker the results in each category.

Variant No.	Simulation Concrete	Simulation PLA	Test PLA
Variant 1	6	6	7
Variant 2	4	4	3
Variant 3	7	7	8
Variant 4	5	5	4
Variant 5	8	8	5
Variant 6	1	1	1
Variant 7	2	2	2
Variant 8	3	3	6

## Data Availability

Not applicable.
